# Endoscopic Full-Thickness Resection for the Management of a Polyp in a Patient With Ulcerative Colitis

**DOI:** 10.7759/cureus.24688

**Published:** 2022-05-03

**Authors:** Gianna Baker, Jessica Vadaketh, Gursimran S Kochhar

**Affiliations:** 1 Division of Gastroenterology, Hepatology, and Nutrition, Allegheny Health Network, Pittsburgh, USA; 2 Internal Medicine, Drexel University College of Medicine, Philadelphia, USA

**Keywords:** endoscopic full-thickness resection, endoscopic gastrointestinal surgery, therapeutic endoscopy, tubular adenoma, colon polyp removal, ulcerative colitis (uc), inflammatory bowel disease

## Abstract

Endoscopic full-thickness resection (EFTR) is an endoscopic technique utilized to excise challenging gastrointestinal lesions. While the safety and efficacy of EFTR are well-documented in the general population, its utilization in patients with inflammatory bowel disease (IBD) has not been reported. Here, we present a patient with a longstanding history (more than 10 years) of ulcerative colitis (UC) who was recently found to have a large, fibrotic, non-lifting adenoma in her descending colon. After a multidisciplinary discussion, it was determined that the best way to remove the adenoma would be by EFTR. To our knowledge, this is the first reported case that details the use of EFTR in a patient with IBD. The procedure was successful, and the patient did not experience any complications during the procedure or upon clinical follow-up.

## Introduction

Endoscopic full-thickness resection (EFTR) is an endoscopic resection procedure currently being used in the removal of lesions at the appendiceal orifice, non-lifting polyps, submucosal lesions, and lesions with significant fibrosis [1]. This technique involves using an over-the-scope clip device (Ovesco, Tübingen, Germany), which aids in retracting the target lesion into the lumen of the cap to allow for the secure fixation of two serosal surfaces after the clip deployment, which isolates the lesion of interest. This lesion can then be resected above the serosal closure with a snare [1].

Many studies have found EFTR to be safe and effective. A recently published meta-analysis of 18 studies comprising 730 patients who had undergone EFTR found that this procedure is a feasible technique for colorectal adenomas, early carcinomas, and gastrointestinal subepithelial lesions [2]. Specifically, the overall pooled rate of complete histological resection was 82%, and the overall pooled rate of en-bloc resection was 95% [2]. Additionally, the rates of post-procedure perforation and bleeding were 0% and 2% [2]. A case series by van der Spek et al. analyzed 51 EFTR procedures in 48 patients and found similar results [3]. The technical success rate was 88%, perforation occurred in one patient (2%), and emergency surgery was not required in any patients [3]. However, six patients (13%) experienced adverse events within 30 days post-procedure [3]. This group of researchers also observed residual or recurrent lesions in five patients (12%) at the surveillance endoscopy 130 days later [3]. Another prospective multicenter study on the safety and efficacy of EFTR in patients with difficult adenomas, early cancers, and/or subepithelial observed a technical success rate of 89.5% with an adverse event rate of 9.9% [4]. Therefore, it is reasonable to suggest that EFTR is a safe and effective treatment option for individuals with colorectal adenomas, early carcinomas, and/or subepithelial lesions.

While extensive research has been done to analyze the safety and efficacy of this therapeutic technique in the general population, there have not been any studies conducted to observe EFTR in patients with inflammatory bowel disease (IBD). However, many studies have analyzed the safety and efficacy of other endoscopic resection procedures, such as endoscopic mucosal resection (EMR) and endoscopic submucosal dissection (ESD), in patients with IBD. These techniques are also used to resect neoplasia but involve resecting from the mucosal and submucosal layers instead of from the muscularis propria [1]. Studies have largely found low rates of perforation, post-procedural bleeding, and the need for surgical intervention with high technical success rates [5-8]. Though these endoscopic resection techniques are safe to perform in individuals with IBD, the frequency of post-procedural recurrence of polyps is higher, and it is more technically difficult to use these techniques to remove a fibrotic, non-lifting adenoma [9-11]. Thus, it was decided to move forward with EFTR in the removal of a fibrotic, non-lifting adenoma in a patient with IBD. Utilizing EFTR over EMR or ESD ensures the complete resection of the adenoma and a reduction in the possibility of polyp recurrence [12].

## Case presentation

**Figure 1:**
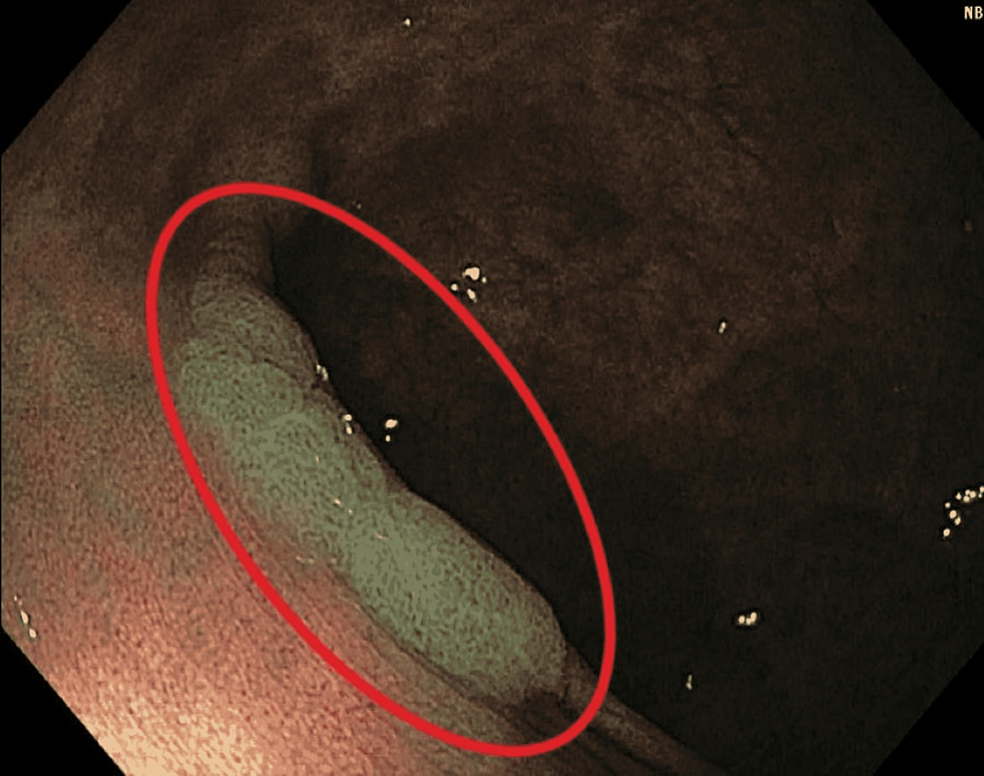
A 30 mm flat, tan-red polyp with no surrounding inflammation in the descending colon

A 76-year-old woman presented to Allegheny General Hospital for an outpatient colonoscopy. The patient had a longstanding history of UC, as well as a known history of colorectal tubular adenomas. At the time of this colonoscopy, she had been off all biologics and immunosuppressants for four years. Additionally, she had been in deep endoscopic and clinical remission. Upon examination, a 25 mm flat, tan-red polyp with no surrounding inflammation was noted in the descending colon (Figure 1).

A polypectomy was not attempted due to the large size and the polyp was not lifting with the lifting solution due to underlying fibrosis.

**Figure 2:**
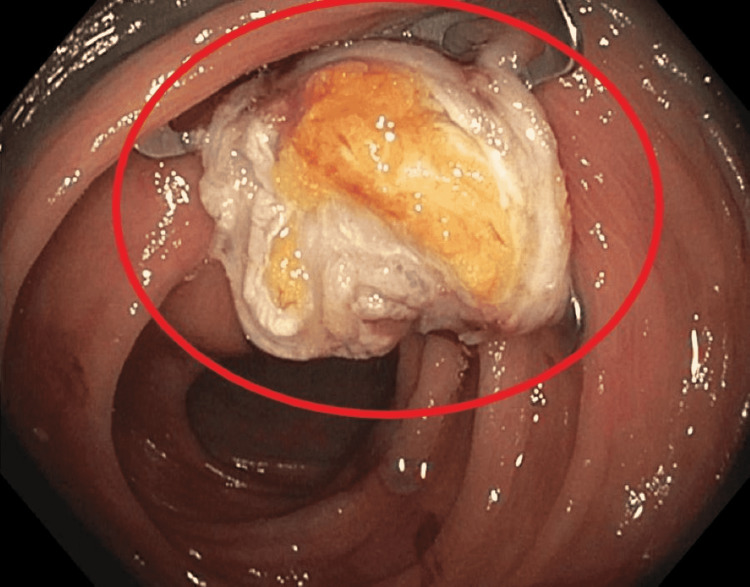
The deployment of the Ovesco surgical clips at the base of the polyp

The patient was scheduled for EFTR using the Ovesco colonic full-thickness resection device (FTRD; Ovesco Endoscopy, Tübingen, Germany). The lesion was marked with the snare tip, and the scope was withdrawn. Then the FTRD device was mounted on the scope and the scope was advanced to the lesion. Using the forceps, the marked area of the polyp was pulled into the FTRD cap. Once the adequate tissue was pulled in, the Ovesco clip was deployed (Figure 2).

**Figure 3:**
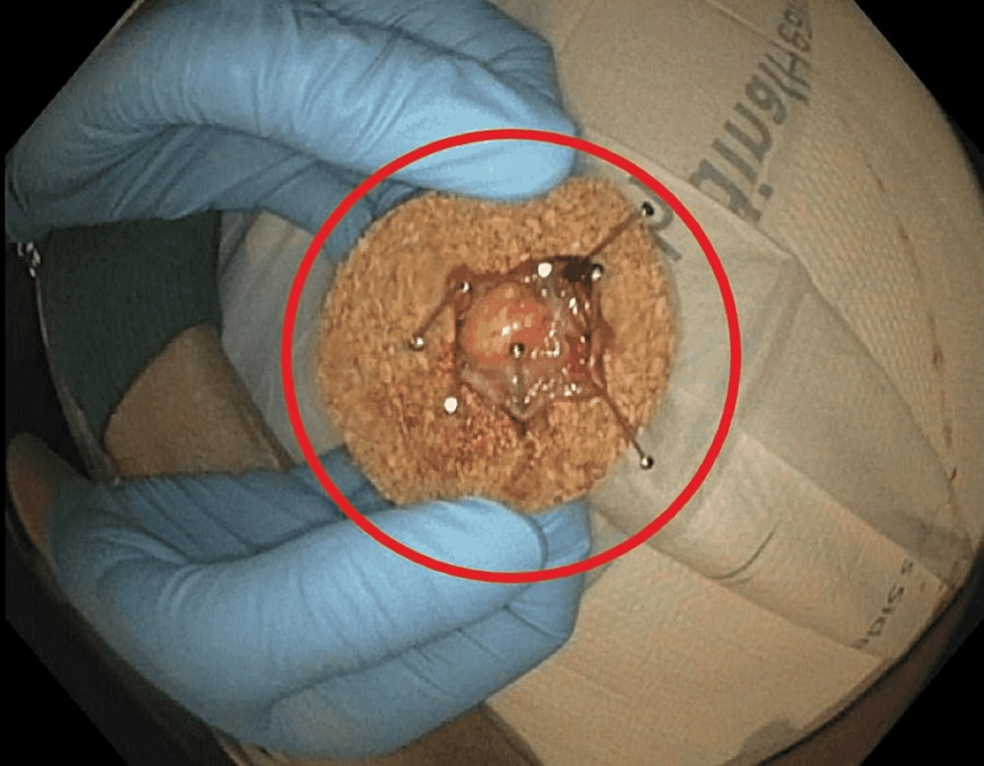
The resected 30 mm polyp

Using the integrated snare, the polyp was cut above the clip and resected (Figure 3).

The post-resection inspection was satisfactory, as there was no bleeding or perforation. The polyp tissue was sent to histology, which later revealed the polyp to be a tubular adenoma. Margins were not involved. The patient was discharged and advised to follow a full liquid diet for two days with a slow re-introduction of food afterward. She was also advised to take 100 mg of Colace for seven days. There were no complications following the procedure.

## Discussion

Ulcerative colitis (UC), a type of inflammatory bowel disease, is a chronic gastrointestinal disorder characterized by inflammation of the colonic mucosa and subsequent mucosal ulceration. This condition requires regular endoscopic surveillance due to the increased risk of developing colorectal cancer from the compounding inflammation, fibrosis, and scar tissue [13]. Specifically, studies have found that the incidence rate of colorectal cancer reaches 18% in patients after 30 years of having UC [14]. Thus, it is recommended to perform a screening colonoscopy eight years following a patient’s diagnosis and every couple of years thereafter [13].

One of the types of precancerous lesions surveilled for on screening colonoscopies is tubular adenomas [15]. This type of benign adenomatous polyp of the colon has the potential to transform into malignancy if it goes undetected [15-16]. Approximately 5% of adenomas transform into cancer [15]. A tubular adenoma is considered high risk if it is larger than 10 mm, villous in nature, or has high-grade dysplasia [16]. Due to the increased risk of colorectal cancer developing from tubular adenomas, it is recommended that these adenomas be removed via endoscopic therapy.

Therefore, our patient’s history of UC and tubular adenomas put her at a higher risk for the progression of a polyp to colorectal cancer. Due to the polyp’s large size and underlying scar tissue, it would have been difficult to completely remove using conventional approaches such as EMR and ESD. To the best of our knowledge, there are no previous reports of EFTR use in IBD patients. However, it has been an effective procedure for removing adenomas and subepithelial and precancerous lesions in non-IBD patients [2]. This case highlights the importance of utilizing EFTR in patients with IBD, as it has the advantage of resecting deeper than other commonly used resection techniques. Here, we demonstrate EFTR as another option for managing colorectal polyps in patients with IBD with challenging polyps.

## Conclusions

This case has demonstrated the first successful utilization of EFTR in a patient with a history of UC, illustrating its therapeutic potential in individuals suffering from UC and polyps. It also signifies the importance of considering the application of EFTR for inflammatory, fibrotic polyps in patients with IBD. EFTR achieves a deeper resection of the lesion, reducing the chances of a residual polyp being left behind and decreasing the risk of the polyp progressing to neoplasia. While no complications were experienced in this case, physicians should carefully consider their patient's candidacy for this treatment based on the patient's history, current endoscopic assessment, and other health implications, which may put the patient at risk for excessive bleeding or perforation.
